# Clindamycin Phosphate Absorption from Nanoliposomal Formulations through Third-Degree Burn Eschar

**Published:** 2015-07

**Authors:** Azadeh Ghaffari, Ali Manafi, Hamid Reza Moghimi

**Affiliations:** 1 Department of Pharmaceutics, School of Pharmacy, Shahid Beheshti University of Medical Sciences, Tehran, Iran;; 2 Department of Food and Drug Control, School of Pharmacy, Zanjan University of Medical Sciences, Zanjan, Iran;; 3 Department of Plastic Surgery, Iran University of Medical Sciences, Tehran, Iran

**Keywords:** Burn eschar, Absorption, Liposome, Clindamycin, Deposition

## Abstract

**BACKGROUND:**

It has been shown that topical nanoliposomal formulations improve burn healing process. On the other hand, it has been shown that liposomal formulations increase drug deposition in the normal skin while decrease their systemic absorption; there is not such data available for burn eschar. Present investigation studies permeation of clindamycin phosphate (CP) through burn eschar from liposomal formulations to answer this question. In this investigation, permeation of CP through fully hydrated third-degree burn eschar was evaluated using solution, normal nanoliposomes and ultradeformable nanoliposomes.

**METHODS:**

Liposomal CP were prepared by thin-film hydration and characterized in terms of size, size distribution, zeta potential, encapsulation efficiency and short-time stability. Then the effect of liposomal lipid concentration on CP absorption was investigated.

**RESULTS:**

The permeability coefficient ratio (liposome/solution) and permeation lag-time ratio (liposome/solution) of CP through burn eschar at 20 Mm lipid concentration were 0.81±0.21 and 1.19±1.30 respectively, indicating the retardation effects of liposomes. Data also showed that increasing liposomal lipid concentration from 20 to 100 mM, clindamycin permeation decreased by about 2 times. There was no difference between normal liposome and ultradeformable liposome in terms of clindamycin absorption.

**CONCLUSION:**

Nanoliposomes could decrease trans-eschar absorption of CP, in good agreement with normal skin data, and might indicate CP deposition in the eschar tissue.

## INTRODUCTION

Burn is one of the most devastating conditions in emergency medicine worldwide involving all age groups and both genders, leading to physical and psychological scars and chronic disabilities^1^ and making patients susceptible to microbial colonization, infection, morbidity and mortality.^2^^,^^3^ Burn injuries during pregnancy are even more dangerous resulting into higher mortality and morbidity in both mother and infant.^4^ For survivors, scarring was demonstrated as the most persisting problem because burn wound healing is a complex process.^5^

For alleviation of such problems, topical antimicrobial therapy remains to be the most important method of wound infection prevention. These compounds need to penetrate the burn eschar for their therapeutic effect. Silver sulfadiazine was shown as the gold standard in topical burn therapy due to its antibacterial properties.^6^^,^^7^ Therefore, introduction of new agents for treatment of burn wounds seems necessary in health care systems with less adverse problems and better efficacy.^8^^,^^9^

Previous studies have shown that most of antibiotics, when applied topically in burn patients, could not penetrate the burn eschar in therapeutic amoumts.^10^ To overcome this problem and enhance drug permeation through burn eschar, different penetration enhancers like terpenes,^11^ surfactants^11^^,^^12^ and even enzymes such as trypsin^13^ have been employed over the past years. Also, our previous studies have shown that hydration state affects drug permeation through burn eschar to a great extent^12^^,^^14^^,^^15^ and that permeation of both hydrophilic and lipophilic drugs through third-degree burn eschar was increased in fully hydrated barrier.^15^ The burn eschar is a proteinous structure with some lipid component.^16^ Hydration changes proteinous structure of burn eschar and it has been shown that pores play an important role in permeation of both clindamycin phosphate (a hydrophilic drug model) and diazepam (a lipophilic drug model) through third-degree burn eschar; it seems that permeation reduces in dehydrated system due to reduced the porosity of the pathway.^15^

Besides, increasing the permeation of drugs through burn eschar and drug delivery beneath this carrier, retention of drugs inside burn eschar is also important. Eschar seems to be a porous structure and may be used as a place to depot drug formulations. Lipid content of burn eschar is decreased in comparison to normal skin. These lipids act as an intercellular mortar and are very crucial for skin barrier properties. Among different delivery systems, liposomes have both properties (depot and bilayer mortar) and are expected to increase or decrease penetration based on mechanism of action.^17^

Nano-particles look promising as new drug delivery systems. However permeation of nano-particles, including liposomes, through intact skin is still controversial and there is not much data available on permeation of liposomes through burn eschar, the subject of the present investigation. Many researchers have shown that liposomes improve drug deposition within skin at the site of action and therefore, reduce drug systemic absorption.^18^ It has been shown that topical liposomal formulations of usnic acid,^19^ basic fibroblast growth factor^20^ and epidermal growth factor^21^ improve second or deep-second burn healing process in rats but, no permeation through eschar were reported. Also, topically applied liposome encapsulated super oxide dismutase reduced post burn wound size and edema formation of deep second-degree burn in rabbit again,^22^ without penetration data. Price et al. showed that tobramycin from topically applied liposomes on full thickness burn eschar in rat remains in high local concentrations with little systemic absorption at 24, 48 and 72 hours post burn.^23^

In the present investigation, permeation of clindamycin phosphate through fully hydrated human burn eschar in the form of normal liposome and ultradeformable liposome was investigated and compared with clindamycin solutions. Clindamycin phosphate was chosen as a hydrophilic drug model with molecular weight of 504.9 and log octanol-water partition coefficient of 0.5. To the best of our knowledge there is no data available in the literature about studying drug absorption through excised human burn eschar from liposomal formulation.

## MATERIALS AND METHODS

Clindamycin phosphate was purchased from Suzhou Pharmaceutical Factory (Suzhou, China). Monobasic potassium phosphate (98-100.5%), phosphoric acid (85-88%), sodium hydroxide (>98%), Tween 80 (for synthesis), triton X-100 (99%) and high performance liquid chromatography HPLC- grade acetonitrile were purchased from Merck (Germany). Sephadex G-50 was purchased from Sigma-Aldrich (England). Egg phosphatidyl cholin (E80) was purchased from Lipoid (Germany). Cholestrol (99%) was purchased from Aldrich (USA).

Liposomal systems (normal liposomes and ultradeformable liposomes) were prepared by the lipid film hydration method.^24^ Briefly, the lipid mixture of the desired molar composition was dissolved in chloroform/methanol (2:1) and dried under reduced pressure in a rotary evaporator at 60°C to form a thin lipid film. The film was then hydrated with clindamycin solution (63 mg/ml) in phosphate buffer solution pH=7.4 at 60°C for 1 hour.^25^ The obtained multivesicular suspensions were extruded (Northern Lipids Inc., Vancouver, Canada) twice through 200 nm and three times through 100 nm pore size Nucleopore polycarbonate membrane filters (Whatman, UK) to produce samples with an appropriate size. 

Different formulation of low lipid, high lipid, purified, empty and normal and ultradeformable liposomes were prepared in this investigation (Table 1). Liposome P100 and ultradeformable liposome was purified from free drug by cellulose dialysis tubing method^26^ before permeation studies. For this purpose, cellulose dialysis tubing with molecular weight cut off of 12 kDa was used and liposome dispersions were placed in donor chamber and dialyzed against releasing medium (phosphate buffer solution, pH=7.4) at 8°C and 300 rpm magnetic stirring for 12 hours. Purified liposomes were stored in refrigerator and used for permeation studies 24 hours later. Liposomes were characterized in terms of size, size distribution, zeta potential, encapsulation efficiency, short-time stability.

Size and size distribution of liposomes were determined by laser diffraction method using a Mastersizer 2000 (Malvern Instruments, London, England). Results are presented as a median diameter of the liposome suspension (d(0.5)) and size distribution are presented by span. Span is the measurement of the width of the distribution. The span was calculated using Equation 1.

(Equation 1)

Span=(d(0.9)-d(0.1))/d(0.5)

Zeta potentials of the liposomes were determined at room temperature in deionized water with a Malvern Zetasizer Nano ZS (Malvern Instruments, London, England). Free drug was first separated from liposomes by size-exclusion chromatography using a Sephadex G-50 column eluted with phosphate buffer solution pH=7.4. Liposomes were then disrupted as follows and the drug content (by HPLC, as described later) and phospholipid content were evaluated. To disrupt liposomes, triton X-100 was added to the liposomes at a final concentration of 1% and vortexed for 5 minutes and centrifuged at 9000×g for 10 minutes.

Phospholipid content of the purified liposome was also determined using the method described by Stewart.^27^ The same experiments were performed on initial liposomes (before separation of free drug) as well. Encapsulation efficacy (EE) was then calculated using Equation 2 in which D/L is drug/lipid ratio (w/w).

(Equation 2) $$\mathrm{EE}\left(\%\right)=\frac{\mathrm{Drug}/\mathrm{Lipid ratio in purified liposome}}{\mathrm{Drug}/\mathrm{Lipid ratio in initial liposome}}\times 100$$

Stability of liposomes in term of EE was studied for one week. Prepared liposomes immediately after preparation were purified from free drug by size exclusion chromatography using a sephadex G-50 column and stored in refrigerator (2-8 ºC). EE of purified liposomes at 1, 2, 5, 7 days were determined as explained above.

Clindamycin solutions were prepared by dissolving clindamycin in buffer phosphate solution (pH=7.4) at similar concentration as that of liposomes. Third-degree burn eschar samples, which were separated at the time of surgical debridement (1–2 weeks post-burn) from burned patients, were obtained from Motahari Burn Center (Tehran, Iran). The cause of burning in all patients was flame. Eschar samples were from 14 patients, 11 men and 3 women (39±18 years, mean±SD). Large pieces of eschar tissues were stored at -20°C until use; not later than 12 months. There is no published data available regarding the effect of freezing on the barrier properties of burn eschar. However, it has been shown that storage of normal human skin at −20◦C, even up to 15 months, does not change its permeability to water.^28^ Besides, preliminary comparison of our data collected over last 2-3 years shows that the length of storage at -20°C does not affect the barrier properties of burn eschar.

For permeation studies, the large pieces of burn eschar were thawed at ambient temperature and cut into appropriate smaller pieces. The eschar samples were fully hydrated by water by placing the samples in water for 12 h at ambient temperature. Permeation studies were performed by home-made diffusion cells with effective surface area of approximately 1.8 cm^2^. Eschar samples were placed between donor and receptor chambers of the cells while the epidermal side faced the donor compartment. One milliliters of clindamycin liposomes or clindamycin solutions was placed in donor chamber and receptor chamber was filled with 25 ml of phosphate buffer solution (pH=7.4).

The cells were then placed in a thermostatically controlled water bath with stirrer. The temperature was kept at (37°C±0.5) in the receptor chamber that gives a temperature of approximately 32°C at the surface of the eschar. The speed of stirring in the receptor chamber was 300 rpm. Serial samples were collected from the receptor chamber for 12 h and their drug contents were analyzed by HPLC. Sink condition was maintained throughout the experiments. The cumulative amount of permeated drug was plotted against time and the slope of the linear portion of the plot was measured as the steady state flux (*J*). Permeability coefficient (K_p_) was then calculated using *J *and donor-drug concentration (C) using Fick’s law (*K*_p_=J/C).

Permeation lag-time and permeability coefficients of clindamycin absorption through burn eschar from solution and liposomes were statistically compared using t-test. The level of significance was set at *p<*0.05. The statistical analysis was computed with the SPSS software version 17.0 (SPSS Inc., Chicago, IL). Also, K_p_ ratio and permeation lag-time ratio (liposome/solution) were calculated and compared in liposomal formulations. To investigate the effect of lipid concentration on clindamycin absorption through burn eschar, clindamycin liposome with lipid concentration of 20 and 100 mM were used without separation of free drug. These experiments used clindamycin concentration of 63 mg/ml. Permeability coefficient (K_p_) ratios, liposomal formulations to solutions K_p_s and also lag-time ratios, liposomal formulations / solutions lag time ratios, were then calculated.

In another part of this investigation, the effect of bilayer flexibility on permeation of clindamycin was investigated. These experiments employed two different liposomes of P100 (normal liposome) and ultradeformable liposome (see Table 1 for details). Data were then compared to clindamycin solutions which were contained clindamycin as exactly same as lipomes. Ultradeformable liposomes are special kind of liposomes which consist of phosphatidyl choline and edge activator. An edge activator is often a single chain surfactant that destabilizes the lipid bilayer of the vesicles and increases the deformability of the bilayer. Studies have shown that ultradeformable liposomes enhance drug permeation through skin more efficiently than normal rigid liposomes.^29^ In current study, Tween 80 was used as edge activator and clindamycin absorption through burn eschar from normal liposome and ultradeformable liposome were investigated. 

**Table 1:** Liposome formulations used in this study

**Liposome code**	**Lipid concentration (mM)**	**Lipid composition**	**Purification method**
Liposome 20	20	EPC/Chol (0.8:0.2 molar ratio)	-
Liposome 100	100	EPC/Chol (0.8:0.2 molar ratio)	-
Liposome P100	100	EPC/Chol (0.8:0.2 molar ratio)	Dialysis
Ultradeformable	100	EPC/Tween 80 (85:15% w/w)	Dialysis

HPLC analysis of clindamycin phosphate was measured by a HPLC method suggested by the United States Pharmacopeia (USP, 2000). Samples were analysed by HPLC apparatus (Merck, Germany), using a 25cm×4.6 mm RP-18 column with 3 μm particle size (Perfectsil Target ODS-3, MZ-Analysentechnik). The mobile phase was acetonitrile and pH=2.5 phosphate buffer (22.5:77.5, v/v). The flow-rate was 1 ml/min, and clindamycin phosphate was detected using a UV detector at a wavelength of 210 nm. The results showed a linear relationship (*r*^2^=0.995) between area under the curve and the concentration of clindamycin phosphate in the range of 0.01–10 mg/ml. Recovery percentage and inter-day and intra-day studies showed good accuracy and repeatability of this method.

## RESULTS

The mean particle sizes, d (0.5), of different liposomal populations (batches) for normal liposomes were in the range of 131-142 nm, with a span lower than 0.88. For the ultradeformable liposomes, the mean particle sizes were in the range of 123-124 nm in different populations, with a span of lower than 0.83. The zeta potentials of both liposomes were found to be in the range of -10.9 to -11.4 mV.

Encapsulation efficiencies (EE) of liposome 100 and ultradeformable liposome were calculated to be 20.8% and 20.3% respectively (Table 2). Entrapped clindamycin (EC) in liposome P100 (purified liposome) was decreased from 20.8% at time zero to 11.4% after one day that means about 50% of drug was released during the 1st day. But, there was no further decrease in EE beyond this time up to 7 days. This might be due to loss of surface-bound drugs in the first day or partial unstability of liposomes. EE of clindamycin in ultradeformable liposome was decreased from 20.3% at time zero to 0.8% after one day indicating that 95% of drug was released during the 1st day (Table 2). These data show that liposome P100 is more stable than ultradeformable liposome in term of drug release. Based on these data, for permeation studies, it was considered here that 50% and 99% of CP was free in liposome P100 and ultradeformable liposome respectively.

**Table 2:** Encapsulation efficiency (EE) of liposomes studied for 1 week after preparation

**Time (day)**	**Liposome P100**		**Ultradeformable liposome**	
	**EE (%)**	**Ratio** a	**EE (%)**	**Ratio** a
0	20.8	1	20.3	1
1	11.4	0.55	0.8	0.04
2	11.4	0.55	0.6	0.03
5	10.8	0.52	0.6	0.03
7	12.1	0.58	0.6	0.03

a In comparison to time zero

**Fig. 1:**
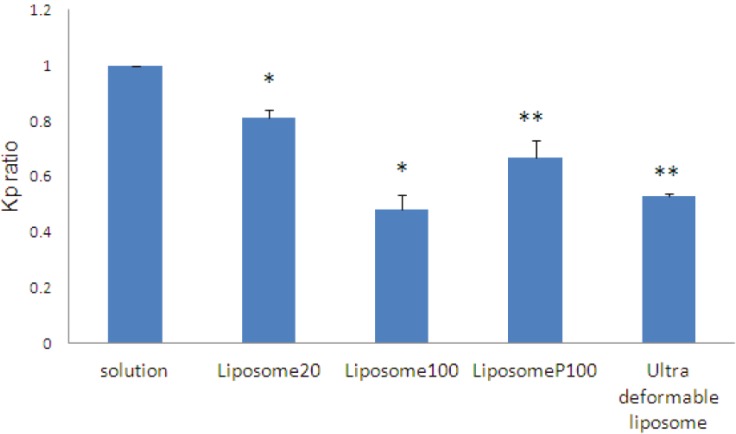
Permeability coefficient (Kp) ratio (liposomal formulation/solution). Data are mean+SEM, n=3-7. Statistical comparisons in t-test for *p*<0.05 are shown as ^*^(Non significant) and ^**^(Significant).

**Fig. 2:**
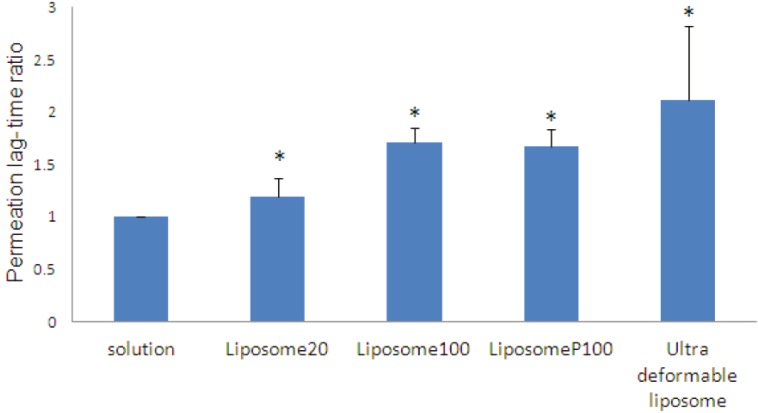
Permeatiom lag-time ratio (liposomal formulation/solution). Data are mean+SEM, n=3-7. Statistical comparisons in t-test for *p*<0.05 are shown as ^*^(Non significant).

The permeability coefficient and permeation lag-time of CP permeated through burn eschars from CP solutions in different sets of experiments were calculated to be in the range of 4.23-1.48×10^-3^ cm/hr and 0.6-1.87 hr respectively. These ranges are broad because CP absorption tests were done on the samples that were obtained from different burned patients and also from different place of burning in one patient. So, differences between K_p_ and permeation lag-time of different eschars are due to biological variations of burn eschars. Therefore, in this study, to reduce the effect of such unavoidable variations, control (CP solution) and test (liposomal formulation) study of each set of experiment were performed on eschar samples prepared from a single eschar tissue. Even with such considerations, CV% of permeation parameters is relatively high but, still acceptable considering the complexity of burn eschar barrier (Table 3). Permeability coefficient (K_p_) ratios, liposomal formulations to solutions K_p_s and also lag-time ratios, liposomal formulations / solutions lag time ratios, are shown in Table 3 and Figure 1 and 2.

**Table 3:** Permeability coefficient (K_p_) ratio and lag-time ratio (liposomal formulation/solution). Data are mean±SD, n=3-7.

Liposomal formulations	(K_p_) ratio	Lag-time ratio
Liposome 20	0.81±0.21*	1.19±1.30*
Liposome 100	0.48±0.33*	1.71±0.85*
Liposome P100	0.67±0.25**	1.67±0.69*
Ultra deformable liposome	0.53±0.03**	2.12±2.09*

* Non significant (liposome and solution; t-test);

** Significant (liposome and solution; t-test)

## DISCUSSION

The results show that liposomal formulations have reduces permeation of CP through burn eschar. As described later this could be due to entrapment of CP in the eschar by liposomes or increased barrier properties of the eschar due to presence of liposomes. These data are in good agreement with decreased permeation of liposomal tobramycin through full thickness burn eschar in rat.^23^

To investigate the effect of lipid concentration of liposome on permeation of clindamycin through burn eschar, clindamycin absorption from liposome 20 (20 Mm lipid) and liposome 100 (100 mM lipid) was studied and compared. K_p_ and permeation lag-time of CP from solution was measured to be 9.26±2.77×10^-3^ cm/hr and 0.8±0.28 hr respectively. For liposome 20 formulation, K_p_ ratio and permeation lag-time ratio (liposome 20/solution) was calculated to be 0.81 and 1.2 respectively (Table 3, Figure 1 and 2). These data show that clindamycin permeability coefficient from liposome 20 was decreased by about 1.2 times and its lag-time was increased about 1.2 times in comparison to clindamycin solution but, the differences are not significant.

By increasing liposomal lipid concentration from 20 to 100 mM, CP K_p_ and permeation lag-time in the set of experiment from solution was 4.23±2.31×10^-3^ and 1.87±0.68 hr respectively. For liposome 100, K_p_ ratio and permeation lag-time ratio (liposome 100/solution) calculated to be 0.48 and 1.71 respectively (Table 3, Figure 1 and 2). These data shows that by increasing liposomal lipid concentration from 20 to 100 mM, clindamycin permeation flux and permeability coefficient were further decreased by about 2 times and its lag-time ratio was further increased from 1.19 to about 1.7 times. These data show that increased lipid concentration decreases permeation of clindamycin through burn eschar and increases its permeation lag-time. So, it might be deducted that by increasing lipid concentration of liposome, deposition of liposomes in eschar pores and subsequently barrier properties of burn eschar was increased. Deposition of liposomal formulations and increased barrier properties for porous membranes (such as filter) has been reported previously for skin models.^30^ They also have shown that by increasing the lipid concentration, the number of bilayers and hence its barrier property increases.^30^

Liposome 100 shows a decreased K_p_ (about 2 times) and increased lag-time (about 1.7 times) in comparison to solution. This might be attributed to clindamycin entrapment in liposome 100 or deposition of liposome in the eschar pores and therefore increased barrier properties of the membrane. As was shown earlier, only about 20% of clindamycin is entrapped in liposome 100 and about 80% of drug is free while, the decrease in flux is about 50%. This clearly shows that increased barrier property of the eschar due to the deposition of liposome is very important in this decrement. 

In liposome P100, 50% of CP was entrapped inside liposome and 50% of CP is free in liposome P100. However, the K_p _ratio (1.5) and permeation lag-time ratio (1.7) are comparable to that of liposome 100 (Table 3, Figure 1 and 2). So, it might be concluded that decreased permeation of CP from liposome 100 and liposome P100 as compared to CP solution is mainly caused by liposome deposition in burn eschar tissue and pores creating a new barrier within eschar. Such a mechanism does not contradict entrapment of drugs in the burn wound after application of liposomes as reported before. They concluded that liposomal tobramycin causes local deposition in the full thickness burn wound with little systemic absorption.^23^

To investigate the effect of bilayer deformability on clindamycin absorption, clindamycin absorption from ultradeformable liposome was also investigated. K_p_ and permeation lag-time of CP from ultradeformable liposome was 16.49±0.97×10^-3^ cm/hr and 2.42±2.22 hr respectively, resulting in K_p_ ratio and lag-time ratio of 0.53 and 2.12 respectively (Table 3, Figure 1 and 2). Ninety nine percent of clindamycin in ultradeformable liposome is free that is 2 times of liposome P100, while permeation flux and permeability coefficient are higher in ultradeformable liposome. So, these data might show that pore closing ability of liposomal formulations is more probable for ultradeformable liposomes in comparison to normal liposomes. This could be due to their flexibility and permeation to deeper parts of the tissue. 

It has been shown in many studies that different drug encapsulated liposomes are effective in drug delivery to burn eschar and improve burn healing process.^19^^-^^22^ Our study also, shows that liposomal formulations deposit on burn eschar and reduce trans-eschar absorption. It might be deducted that deposition of liposomes in the burn eschar increases drug deposition in the eschar. In this direction it has been shown that tobramycin from topically applied liposomes on full thickness burn eschar in rat remains in high local concentrations with little systemic absorption at 24, 48 and 72 hours post burn^17^, in good correlation with the present study and discussion. 

## Note

Please cite this paper as:

Ghaffari A, Manafi A, Moghimi HR. Clindamycin Phosphate Absorption from Nanoliposomal Formulations through Third-Degree Burn Eschar. World J Plast Surg 2015;4(2):145-152.
